# Search for genetic variants in the p66^Shc ^longevity gene by PCR-single strand conformational polymorphism in patients with early-onset cardiovascular disease

**DOI:** 10.1186/1471-2156-7-14

**Published:** 2006-03-06

**Authors:** Federica Sentinelli, Stefano Romeo, Fabrizio Barbetti, Andrea Berni, Emanuela Filippi, Marzia Fanelli, Mara Fallarino, Marco G Baroni

**Affiliations:** 1 Department of Medical Sciences, Endocrinology, University of Cagliari, Cagliari, Italy; 2 Department of Clinical Sciences, Division of Endocrinology, University of Rome "La Sapienza", Rome, Italy; 3 IBCIT, Biomedical Scientific Park of Rome S Raffaele, Rome, Italy; 4 Department of Cardiology, II Faculty of Medicine, University of Rome "La Sapienza", Rome, Italy

## Abstract

**Background:**

Among the possible candidate genes for atherosclerosis experimental data point towards the longevity gene p66^Shc^. The p66^Shc ^gene determines an increase of intracellular reactive oxygen species (ROS), affecting the rate of oxidative damage to nucleic acids. Knock-out p66^Shc-/- ^mice show reduction of systemic oxidative stress, as well as of plasma LDL oxidation, and reduced atherogenic lesions. Thus, p66^Shc ^may play a pivotal role in controlling oxidative stress and vascular dysfunction *in vivo*.

**Methods:**

We searched for sequence variations in the p66^Shc ^specific region of the Shc gene and its upstream promoter by PCR-SSCP in a selected group of early onset coronary artery disease (CAD) subjects (n. 78, mean age 48.5 ± 6 years) and in 93 long-living control subjects (mean age 89 ± 6 years).

**Results:**

The analysis revealed two variant bands. Sequencing of these variants showed two SNPs: -354T>C in the regulatory region of p66^Shc ^locus and 92C>T in the p66 specific region (CH2). Both these variants have never been described before. The first substitution partially modifies the binding consensus sequence of the Sp1 transcription factor, and was detected only in two heterozygous carriers (1 CAD subjects and 1 control subject). The 92C>T substitution in the CH2 region consists in an amino acid substitution at codon 31 (proline to leucine, P31L), and was detected in heterozygous status only in one CAD subject. No subjects homozygous for the two newly described SNPs were found.

**Conclusion:**

Only two sequence variations in the p66^Shc ^gene were observed in a total of 171 subjects, and only in heterozygotes. Our observations, in accordance to other studies, suggest that important variations in the p66^Shc ^gene may be extremely rare and probably this gene is not involved in the genetic susceptibility to CAD.

## Background

Increasing evidence indicates that reactive oxygen species (ROS) may participate in the pathogenesis of various diseases, including cardiovascular disorders. Support to this comes from the experimental demonstration that vessel walls of patients with atherosclerotic risk factors are characterized by a significant increase in vascular ROS production [1].

It has been reported that the p66^Shc ^longevity gene increases intracellular reactive oxygen species (ROS), thereby affecting the rate of oxidative damage to nucleic acids [2]. The human Shc locus (Src homologous and collagen) encodes three proteins with relative molecular masses of 46K (p46^Shc^), 52K (p52^Shc^) and 66K (p66^Shc^). All three proteins share a Src-homology2 (SH2) domain, a collagen-homology (CH1) region and a phosphotyrosine-binding (PTB) domain. The p66^Shc ^protein contains a unique amino terminal region (CH2) [3]. p46^Shc^, p52^Shc ^and p66^Shc ^are adaptor proteins in the insulin-signalling pathway, but their downstream effects differ: p46^Shc ^and p52^Shc ^are tyrosine-phosphorylated by a variety of growth factors, and are associated with MAPK signalling; p66^Shc ^also undergoes tyrosine phosphorylation in response to extracellular signals but is involved in signal transduction pathways that inhibits the activation of c-fos promoter [4]. C-fos is transcriptionally activated in response to environmental stresses such as ultraviolet light (UV) or H_2_O_2_. Thus, there is *in vitro *evidence that p66^Shc ^is part of a complex transduction pathway that controls oxidative stress [4].

Knock out mice for the p66^Shc ^gene locus show a higher resistance to oxidative stress and extended life span (up to 30%) [3]. Moreover, p66^Shc-/- ^cells show a reduced oxidative stress-induced apoptosis, which is restored by p66^Shc ^over expression [3].

Also, the role of p66^Shc ^in muscle damage that follows acute hindlimb ischemia has been investigated. Results showed that p66^Shc-/- ^mice were resistant to tissue damage induced by ischemia and ischemia/reperfusion secondary to ROS generation, demonstrating that p66^Shc ^plays a crucial role in the cell death pathways activated by acute ischemia [5].

Further studies using p66^Shc-/- ^mice showed a significant reduction of systemic oxidative stress, plasma LDL oxidation and early atherosclerotic lesions when mice were fed a high-fat diet [6].

p66^Shc ^expression is tissue specific [7] and it is regulated by epigenetic modifications, namely histone deacetylation and cytosine methylation. In this regard, it has been reported that histone deacetylase inhibitors and demethylating agents restore p66^Shc ^expression in human peripheral blood lymphocytes (PBL) that normally do not express this isoform [8].

More recent studies have observed that absence of p66^Shc ^expression might contribute to the protection of the heart from the deleterious effects of elevated Angiotensis II levels [9]. Furthermore, it was reported that inactivation of the p66^Shc ^gene protects against age-dependent ROS-mediated endothelial dysfunction [10] in p66^Shc ^KO mice.

It appears therefore that p66^Shc ^may play a pivotal role in controlling oxidative stress and vascular dysfunction *in vivo*, possibly regulating the evolution of the atherosclerotic process. Based on this background, we investigated the p66^Shc ^specific region of the Shc gene and its upstream promoter for variations in a selected group of subjects with early-onset coronary artery disease (CAD).

## Results

The possible effect of p66^Shc ^longevity gene mutations on CAD would probably determine an early onset of the disease, and assuming that a genetic effect might be more evident in younger patients, we choose to select subjects with age of diagnosis of CAD <55 years. This age limit was based on the incidence curves of CAD and the estimates of genetic effect at various ages [11,12]. As controls we studied 93 unrelated long-living subjects (mean age 89 ± 6 years) randomly selected from a population of individuals screened for CAD risk factors.

**Figure 1:**
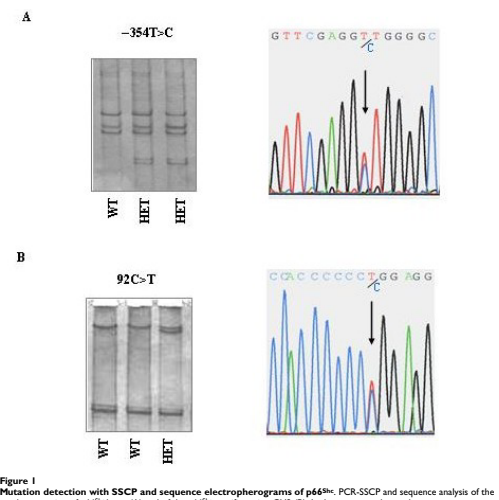
**Mutation detection with SSCP and sequence electropherograms of p66^Shc^**. PCR-SSCP and sequence analysis of the regulatory region of p66^Shc ^locus (A) and of the p66^Shc ^specific region CH2 (B). In the sequence electropherograms, arrows indicate the nucleotide substitution in heterozygous subjects as shown by the presence of the 2 superimposed peaks representing the normal and abnormal base. **A**) SSCP analysis of the 5' regulatory region of p66 locus (*T/T *subjects with wild-type sequence, *T/C *subjects heterozygous for the variant). **B**) SSCP analysis of the CH2 region: the 92C>T substitution consisted in an amino acid substitution P31L (*C/C *subjects with wild-type sequence, *C/T *subject heterozygous for the variant).

The human Shc locus encodes three proteins (p46, p52 and p66) by using two alternative promoters that synthesize two mRNAs coding for p46/p52 and for p66 [8]. The p66^Shc ^promoter, positioned in the first intron of the Shc locus, contains a relative high frequency of CpG dinucleotides whose methylation regulates the transcriptional activity of p66^Shc ^promoter [8]. Considering the recent observations that alterations in expression of p66^Shc ^gene might be one possible cause in determining life span and in controlling oxidative stress, we hypothesised that sequence variations in p66^Shc ^promoter may contribute to both aging and early atherogenesis. PCR-SSCP analysis of the p66 specific region of the Shc locus and the p66^Shc ^promoter (from nucleotide -637) revealed two variant bands (Figure 1). Sequencing of these variants showed two SNPs: -354T>C in the regulatory region of p66^Shc ^locus and 92C>T in the p66 specific region (CH2). The first substitution partially modifies the binding consensus sequence of the Sp1 transcription factor, and was detected only in two heterozygous carriers (1 CAD subject and 1 control subject). The 92C>T substitution in the CH2 region consists in an amino acid substitution at codon 31 in which proline is substituted with leucine (P31L), and was detected in heterozygous status only in one CAD subject. No subjects homozygous for the two newly described SNPs were found. One family member of the subject carrying the P31L mutation, also affected by early-onset CAD (the brother, age 37 years), was screened for these new polymorphisms, but no SNPs were detected.

## Discussion

We screened for new DNA polymorphisms the p66 specific region of the Shc locus (CH2) and the p66^Shc ^promoter. We identified by PCR-SSCP assay of 1167 base pairs in 4 amplified segments two segregating sites in 2 out of the 342 chromosomes examined. The two novel variants (-354T>C and 92C>T) were very rare in both populations of CAD and elderly subjects. Furthermore, no subject homozygous for the two SNPs was found.

Our observations agree with a previous study performed in a sample of extreme survivors of the Leiden 85-plus Study, where no sequence variations in the p66 specific region of the Shc gene were found [13].

The Shc gene contains one known non-synonymous polymorphism located in the CH1 domain which corresponds to Met^300^Val in the p52 isoform and to Met^410^Val in the p66 isoform [13]. No association of the Met^300^Val (the p52 isoform) variation was found with birth weight and length, impaired insulin secretion, insulin resistance and type 2 Diabetes Mellitus in a cohort of 360 young healthy Danish Caucasian subjects [14]. In a more recent study the Met^410^Val variant (the p66 isoform) was tested for association with longevity using a prospective follow-up design in two independent cohorts of 730 and 563 subjects aged 85 and over [13]. Mooijaart and co-workers observed a small increase in Val allele with increasing age of death and a decreased mortality risk of Met/Val carriers compared to Met/Met carriers; however no statistical significance was reached in the two different analyses [13]. These results suggest a possible beneficial effect of the Valine allele on longevity, but no subject homozygous for valine was found and the frequency of this allele was very low in this cohort. To date, no functional studies on the Met/Val variant of the p66^Shc ^isoform have been performed.

Finally, this Met/Val amino acid variant is present in all 3 isoform of the Shc gene, thus suggesting that this polymorphism is unlikely to specifically affect the function of p66^Shc ^and not of p46^Shc ^and p52^Shc^. For all these reasons we did not study the Met/Val variant in our cohorts, since we were looking for variants that would affect particularly the p66^Shc ^gene.

## Conclusion

Longevity is a complex trait, resulting from the interaction between multiple genetic and environmental factors. Before our, several studies in long-lived subjects have highlighted the role of biological factors as determinant of 'successful ageing'. For example associations between successful ageing and paraoxonase 1 (PON1) and interleukin 10 (IL-10) gene polymorphisms have been reported in centenarian [15-17]. However, confirmatory data is needed, and probably several other genetic factors are involved.

In our study we had hypothesised that the p66^Shc ^gene may play a pivotal role in controlling oxidative stress and vascular dysfunction *in vivo*, possibly regulating the evolution of the atherosclerotic process. In view of its association with CAD in animal models, we assumed that sequence variations would be less frequent in elderly survivors compared to younger CAD subjects.

We screened for new DNA polymorphisms the p66 specific region of the Shc locus (CH2) and the p66^Shc ^promoter and identified two very rare novel variants with no subject homozygous for these SNPs.

Our observations agree with a previous study where no sequence variations in the p66 specific region of the Shc gene were found [13]. In the rest of the Shc gene, only one nucleotide substitution (Met/Val) has been reported, located in the CH1 domain and common to all three isoforms of the Shc gene [13,14]. These observations, together with our data, suggest that important variations in the Shc gene may not be tolerated and that evolution might have selected against their occurrence, and it appears that the role of p66^Shc ^gene in mammalian longevity is probably more complex than previously thought.

## Methods

### Subjects selection

The p66 specific region of the Shc locus and the p66^Shc ^promoter were screened for new sequence variants in 78 subjects with early-onset coronary disease (<55 years, mean age 48.5 ± 6 year) recruited among subjects undergoing coronary angioplasty or presenting with clear evidence of CAD (previous diagnosis of MI, or one or more stenoses greater than 50% in at least one major coronary artery after coronary catheterisation and clinical symptoms of angina). Diagnosis of MI was based on typical electrocardiographic changes and elevation of at least two enzymes, and was confirmed by the presence of wall motion abnormalities in left ventriculography.

Control subjects were 93 unrelated long-living subjects (mean age 89 ± 6years) randomly selected from a population of individuals screened for CAD risk factors. Exclusion criteria were: 1) the presence of CAD 2) the presence of type 2 diabetes. CAD was excluded by use of the Rose questionnaire [18] and ECG (Minnesota coding). A complete medical history in all subjects was obtained by questionnaire. History taking included questions about smoking habits, history of hypertension and type 2 diabetes and current medications. Diagnosis of type 2 diabetes was based on history of hypoglycaemic treatment and/or confirmed fasting blood glucose >126 mg/dl (7.0 mmol/L). All Caucasian subjects were recruited in the Centre-West Coast of Italy, most from Rome and its surrounding towns. All subjects gave their written informed consent to their participation to the study. The study was approved by the University of Rome "La Sapienza" Ethical Committee.

### Molecular genetic screening

Sequence variants of the p66^Shc ^gene were analyzed with the PCR-SSCP technique as previously described [19]. Briefly, primers were designed to include the specific amino terminal region (CH2) of the p66 protein and the p66^Shc ^promoter in order to evaluate possible mutations and to yield PCR fragments of 200–350 bp (Table 1). After PCR amplification, DNA fragments were electrophoresed in 1X MDE gel (FMC BioProducts, USA), and visualized with silver staining. All amplicons not showing SSCP variants with this method were electrophoresed in a 12% polyacrylamide gel in 1X TBE buffer and run at room temperature at a constant 25 mA for 14–16 h, with and without 5% glycerol. All SSCP variants were investigated by direct sequencing using the PRISM Dye Terminator Cycle Sequencing kit and an ABI 310 automated sequencer (Applied Biosystems) according to the manufacturer's instructions.

**Table 1:** Primers for PCR-SSCP analysis of p66^Shc ^gene

***Amplicon***	***Product size (bp)***	***Forward primer (5'-)***	***Reverse primer (5'-)***
CH2	352	gcccctctttcacctcaact	atgtcctggaggaggggtag
Promoter-1	238	cagagaagaggctccacgtt	ggggcaggagatccatagtt
Promoter-2	247	cccacccccactttacttct	aacgtggagcctcctctctg
Promoter-3	330	aggacaggaagggaaatgct	agtgctgactcacaggctca

Forward and reverse primer sequences are all 5'. Promoter-1 fragment starts at nucleotide -222 from start codon of exon1; Promoter-2 fragment starts at nucleotide -449 from start codon of exon1; Promoter-3 fragment starts at nucleotide -637 from start codon of exon1 of p66^Shc ^gene.

## Abbreviations

SSCP, single-strand conformational polymorphism; CH2, collagen-like domain at the amino-terminus; MI, myocardial infarction, CAD, coronary artery disease; SNP, single nucleotide polymorphism.

## Authors' contributions

The studies were designed by FS and MGB. All subjects data were obtained by AB, FB, MF and the database organization was carried out by SR and MF. Experimental data was obtained by FS and EF. Data analyses were performed by FS and MGB. The paper was written by FS and MGB and all authors read and approved the final manuscript.
